# P-902. Etiology, Clinical Characteristics, Prognosis and Outcomes of Acute Central Nervous System Infections: A Prospective Study

**DOI:** 10.1093/ofid/ofae631.1093

**Published:** 2025-01-29

**Authors:** Ashwin Pillai, Shea-Lee Godin, Nazir Edul

**Affiliations:** University of Connecticut, Farmington, Connecticut; Resident, Hartford, Connecticut; Ruby Hall Clinic, Pune, Maharashtra, India

## Abstract

**Background:**

Various pathogens can cause central nervous system (CNS) infections. Early recognition and treatment significantly improves outcomes. Clinical features and outcomes vary by etiology and clinical phenotype, making diagnosis challenging. We report a single-center experience emphasizing the clinical profile and prognostic factors of this entity.

**Figure:**
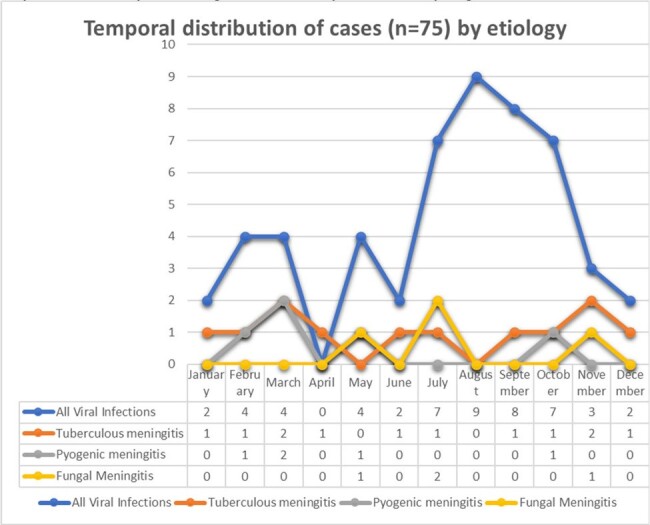

**Methods:**

We conducted a two-year prospective, observational study at a tertiary care center in Western India. Patients suspected to have CNS infections underwent a standardized workup that included neuroimaging and CSF polymerase chain reaction panel testing. Patients were followed throughout the admission. The outcomes recorded were duration of intensive care unit (ICU) stay, hospital stay, modified Rankin Scale (mRS) at discharge and mortality.

**Figure:**
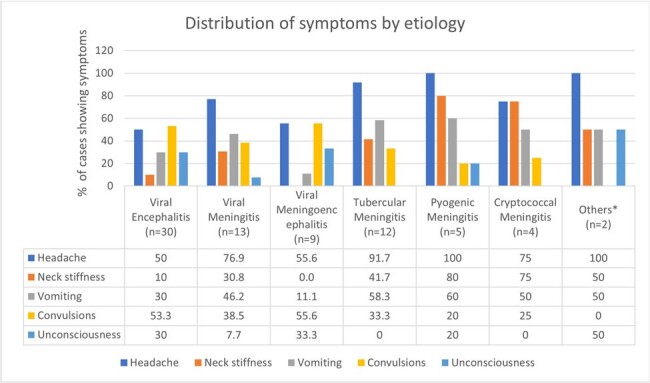

**Results:**

We included 75 patients (53 male, 22 female). The mean age was 43±19 years. Most infections were viral (69%) – 40% encephalitis, 17% meningitis and 12% meningoencephalitis. Viral infections surged in the monsoon and post-monsoon period (Fig. 1). Tuberculous meningitis accounted for 16% cases, pyogenic meningitis for 7%, and cryptococcal meningitis for 5%. Mucor cerebritis and brain abscesses accounted for 3%. Presenting symptoms, CSF, and neuroimaging findings are listed in Fig. 2-4.

Mortality was highest for cryptococcal meningitis (25%); followed by pyogenic meningitis (20%); and viral encephalitis and tuberculous meningitis (16.7% each). Increased risk of mortality was observed with: new-onset seizures (relative risk [RR] 3.58, 95% confidence intervals [CI] 1.03-12.45, p< 0.001); a Glasgow Coma Scale (GCS) < 8 (RR: 6.85, 95% CI 2.00-23.40, p=0.002); and the presence of dilated ventricles on imaging (RR: 5.25, 95% CI 1.99-13.82, p< 0.001).

Increasing age correlated with a longer ICU stay (r=0.37, p< 0.001), hospital stay (r=0.33, p=0.002), and worse functional status at discharge reflected by a higher mRS (p=0.04). GCS at presentation correlated inversely with functional recovery reflected by the mRS at discharge (p< 0.001).

CSF findings varying by etiology

**Figure:**
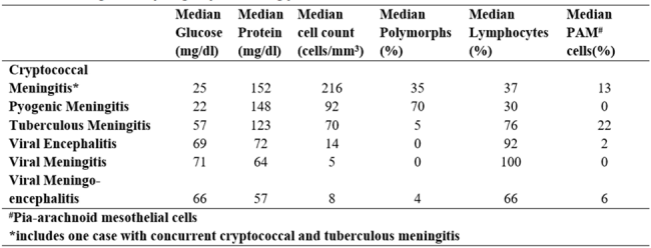

CSF findings varying by etiology

**Conclusion:**

The etiology and prognosis of acute CNS infectious varies by region and season. A detailed history, thorough

physical examination, neuroimaging and CSF analysis are vital as each provides diagnostic and prognostic information.

**Figure:**
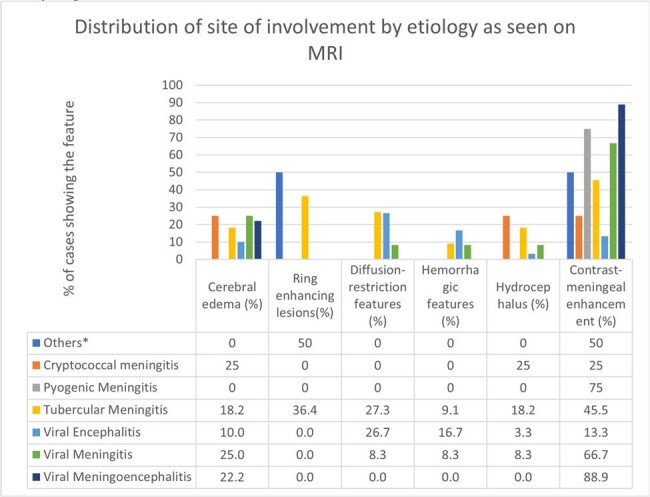

**Disclosures:**

**All Authors**: No reported disclosures

